# Electrical Irritation of Fillings

**Published:** 1897-03

**Authors:** A. H. Porter

**Affiliations:** Philadelphia


### ELECTRICAL IRRITATION OF FILLINGS.

BY DR. A. H. PORTER, PHILADELPHIA.

Volta's contact force and the chemical are the two rival theories that account for the electricity in a battery. To apply these to dental science, the inference from Volta's proposition is that filling and the tooth produce an electrical tension, and that any fluid existing in a crevice reduces the tension. The generally accepted chemical theory asserts that the fluid is both cause of the tension and release. Hence if Volta's be true and no crevice exist, an anæmic tooth is subject to a constant electrical pressure from a filling, which experience proves it will not withstand. Such a force in a stronger tooth will attract a larger blood-supply and build dense dentine.

Metallic fillings excite a greater electrical tension than cement.

Volta's position is reinforced by the well-known experiment of soldering metals and noting the opposite action of each on an electrified needle.

The Austen Roberts experiment is more recent: certain metals when solid diffuse into each other in time when in contact. Whether any dental author has pointed out the above distinction, the present writer has no knowledge. Dr. Palmer's electro-chemical theory draws attention chiefly to the destructive action of fluids electrically decomposed. The facts tend to show that positive electricity is nothing but force in matter of high intensity including light, and that negative electricity is nothing but force of low intensity including heat.

Both light and heat excite electro-magnetic vibration. As electricity ceases to be exhibited in a wire, light and heat appear, showing their identity with electricity.

Light, like positive electricity, discharges negative electricity.

X-ray pictures are taken by the (negative) waves (low vibrations) from a kerosene flame, magnets, ordinary anodes and cathodes, as well as Crookes's tubes.

The well-known experiment of a coin leaving its picture on a highly polished metal surface without pressure and by mere contact is through the same agency.

